# Lack of Vertical Transmission of Severe Acute Respiratory Syndrome Coronavirus 2, China

**DOI:** 10.3201/eid2606.200287

**Published:** 2020-06

**Authors:** Yang Li, Ruihong Zhao, Shufa Zheng, Xu Chen, Jinxi Wang, Xiaoli Sheng, Jianying Zhou, Hongliu Cai, Qiang Fang, Fei Yu, Jian Fan, Kaijin Xu, Yu Chen, Jifang Sheng

**Affiliations:** The First Affiliated Hospital, College of Medicine, Zhejiang University, Hangzhou, China

**Keywords:** Severe acute respiratory syndrome coronavirus 2, SARS-CoV-2, pregnancy, vertical transmission, viruses, coronaviruses, 2019 novel coronavirus disease, COVID-19, China, respiratory infections

## Abstract

A woman with coronavirus disease in her 35th week of pregnancy delivered an infant by cesarean section in a negative-pressure operating room. The infant was negative for severe acute respiratory coronavirus 2. This case suggests that mother-to-child transmission is unlikely for this virus.

The recent outbreak of severe acute respiratory syndrome coronavirus 2 (SARS-CoV-2), which causes coronavirus disease (COVID-19), is a public health emergency that has drawn international concern (*1*). Like SARS-CoV and Middle East respiratory syndrome coronavirus, SARS-CoV-2 is a member of the coronavirus family for which no evidence of mother-to-fetus transmission has been found (*2*–*4*). We report a pregnant woman with confirmed SARS-CoV-2 infection who underwent cesarean section delivery of a SARS-CoV-2–negative infant in Zhejiang Province, China.

On February 6, 2020, a 30-year-old pregnant woman at 35 weeks’ gestation sought treatment at a college hospital because of a 2-day history of dry cough without fever, chills, or shortness of breath. The previous day, she had been confirmed positive for SARS-CoV-2 infection at her local hospital on the basis of a sputum sample.

We analyzed her epidemiologic history by asking questions to determine the timeline of her virus infection. Beginning on January 12, her mother-in-law and father-in-law, who lived in Hubei Province, visited her house for 18 days. After the pregnant woman’s hospital admission, the asymptomatic parents-in-law were suspected of having SARS-CoV-2 infection and therefore quarantined in a hospital. However, they tested negative for SARS-CoV-2 twice, even though radiographs of the lungs of both persons suggested lesions. The pregnant woman’s husband, who denied any contact with others except his wife and his parents, experienced fever on February 1. His condition worsened, and he went to the hospital, where he tested positive for SARS-CoV-2.

On day 1 of her hospitalization, the pregnant woman had dry cough and a temperature of 37.2°C. Chest auscultation was slightly thicker in the right lung but not the left lung. Chest radiography showed scattered multiple patchy infiltrates in both lungs. Laboratory findings were slightly abnormal (Appendix Table 1). We considered this an ordinary case on the basis of mild symptoms and radiologic imaging. She received antiviral treatment (oral lopinavir 200 mg and ritonavir 50 mg, each 2×/d), as well as methylprednisolone (40 mg 1×/d) to relieve inflammation effusion (Figure). Her cough resolved on day 2 of hospitalization.

**Figure:**
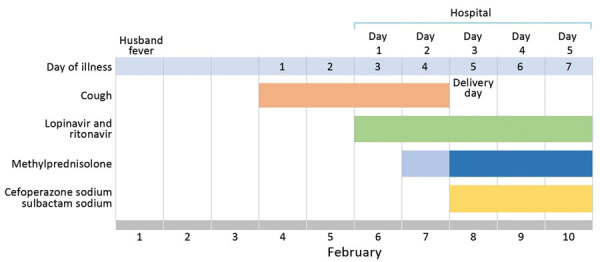
Course of illness and treatment for a 30-year-old pregnant woman infected with severe acute respiratory syndrome coronavirus 2, China.

On February 8 (day 3 of hospitalization), SARS-CoV-2 RNA remained in the woman’s sputum, and the fetal heart rate monitor showed 110 beats/min. Consultation with an obstetrician resulted in a recommendation for emergency cesarean section. The woman underwent a lower uterus cesarean section in a negative-pressure operating room. All persons in the room wore protective suits. Cefoperazone sodium/sulbactam sodium (intravenous drip, 2 g/ 8 h) was infused to prevent infection at the surgical site, and the methylprednisolone dose was doubled. She delivered a normal baby boy without complications. An oropharyngeal swab specimen, obtained immediately after he was taken from the uterus, indicated he was negative for SARS-CoV-2, and he was sent to the negative-pressure ward. During the next 2 days, the infant’s oropharyngeal swab, blood, feces, and urine samples remained negative for SARS-CoV-2 throughout testing at 7 different times.

On the delivery day, although the woman’s sputum was positive, serum, urine, feces, amniotic fluid, umbilical cord blood and placenta, and breast milk samples were negative. On days 4 and 5 of hospitalization, the woman’s sputum tests were negative for SARS-CoV-2, and she remained afebrile. Her respiratory specimens were positive for SARS-CoV-2 after 4 days of serial testing (Appendix Table 2). No SARS-CoV-2 RNA was detected in fecal, urine, and blood samples. The woman was discharged from the hospital on February 19, and her infant was discharged on February 24.

In a descriptive study of SARS-CoV-2 infection, 7 of 9 pregnant women delivered their infants through caesarean section and 2 delivered through vaginal delivery; 10 SARS-CoV-2–negative infants were born (*5*). During caesarean section delivery, N95 mask, goggles, medical protective suit; gentle procedures to avoid droplets from the surgical site; and strict aseptic operation are required. Protective measures are crucial, and the surgery should be performed in a negative-pressure operating room. The virus is now known to interact with human respiratory epithelial cells through its spike protein (*6*). Therefore, it is mainly transmitted through droplets and aerosols.

In conclusion, we report a pregnant woman with SARS-CoV-2 infection who delivered a healthy infant, suggesting that mother-to-child transmission is unlikely for this virus. We believe that effective implementation of protection measures during delivery, including a negative-pressure delivery room, may help prevent the infant from acquiring SARS-CoV-2 infection. Because our conclusions are limited by our sample size of 1, we cannot definitively state whether cesarean section is better than vaginal delivery for preventing transmission from a pregnant mother with SARS-CoV-2 infection.

Appendix Additional test results for pregnant woman with severe acute respiratory syndrome coronavirus 2 and her infant, China, 2020.
