# Supplementary material for: Lack of Vertical Transmission of Severe Acute Respiratory Syndrome Coronavirus 2, China
Source: Emerg Infect Dis. 2020 Jun;26(6):1335–6. doi: 10.3201/eid2606.200287 (PMC7258467; doi:10.3201/eid2606.200287)
Supplement: Appendix — Additional test results for pregnant woman with severe acute respiratory syndrome coronavirus 2 and her infant, China, 2020. [file 20-0287-Techapp-s1.pdf]

Article DOI: https://doi.org/10.3201/eid2606.200287

Lack of Vertical Transmission of Severe Acute Respiratory Syndrome Coronavirus 2, China

Appendix

Appendix Table 1. Laboratory test results for a severe acute respiratory syndrome coronavirus 2–infected pregnant woman during hospitalization, China, February 2020*

Laboratory result (reference range)	Illness day 3, hospital day 1	Illness day 4, hospital day 2	Illness day 5, hospital day 3	Illness day 6, hospital day 4	Illness day 7, hospital day 5
Leukocytes, × 10^9 cells/L (4.0–10.0)	8.7	7.2	8.4	22.9	19.9
Neutrophils, × 10^9cells/L (2.0–7.0)	7.1	5.3	6.9	20.2	17.9
Lymphocytes, × 10^9 cells/L (0.8–4.0	1.1	1.4	1.4	1.9	1.5
Platelets, × 10^9 cells/L (101.0–320.0	96	91	92	108	141
Haemoglobin, g/dL (113.0–151.0)	114	102	106	98	101
Prothrombin time (10.0–13.5)	10.9	–	11.2	10.6	10.4
D-dimer, μg/L (0.0–700.0)	3139	–	–	4784	1836
Urea, mmol/L (2.6–7.5)	–	3.95	3.1	2.84	4.34
Creatinine, μmol/L (41.0–73.0)	–	55	48	49	39
Bilirubin, μmol/L (0.0–21.0)	–	11	8.4	3.9	5.2
ALT, U/L (5.0–40.0)	–	15	9	11	18
AST, U/L (8.0–40.0)	–	19	16	20	18
CK, U/L (38.0–174.0)	–	–	–	62	46
CRP, mg/L (0.0–8.0)	–	–	18.59	14.4	11.17
Lactic acid, mmol/L (0.5–1.6)	–	1.4	0.9	1.5	1.9
IL-2, pg/mL (0–4.13)	–	0.95	–	0.95	–
IL-6, pg/mL (0–6.61)	–	21.93	–	28.48	–
IL-10, pg/mL (0–2.31)	–	2.08	–	1.19	–
TNF-α, pg/mL (0–33.27)	–	6.55	–	6.55	–
IFN-γ, pg/mL (0–20.06)	–	7.35	–	2.41	–

*ALT, alanine aminotransferase; AST, aspartate aminotransferase; CK, creatine kinase; CRP, C-reactive protein; IFN, interferon; IL, interleukin; TNF, tumor necrosis factor; –, not available.

Appendix Table 2. Results of real-time reverse transcription PCR for SARS-CoV-2 in a pregnant woman during hospitalization and in her infant immediately after birth, China, February 2020*

	Mother					Infant	
Specimen	Illness day 3	Illness day 4	Illness day 5	Illness day 6	Illness day 7	Day 1	Day 2
Sputum	Pos	Pos, C_t 30.9	Pos, C_t, 36.8	Pos	Pos	NT	NT
Serum	NT	Neg	Neg	Neg	Neg	Neg	Neg
Urine	NT	Neg	Neg	Neg	Neg	Neg	Neg
Stool	NT	Neg	NT	NT	NT	Neg	Neg
Amniotic fluid	NT	NT	Neg	NT	NT	NA	NA
Umbilical cord blood	NT	NT	Neg	NT	NT	NA	NA
Placenta	NT	NT	Neg	NT	NT	NA	NA
Breast milk	NT	NT	Neg	Neg	Neg	NA	NA

*C_t, cycle threshold; NA, not available; neg, negative test result for SARS-CoV-2; NT, not tested; pos, positive result for SARS-CoV-2. SARS-CoV-2, severe acute respiratory syndrome coronavirus 2.
